# Systematic review and meta-analysis of serotonin transporter genotype and discontinuation from antidepressant treatment

**DOI:** 10.1016/j.euroneuro.2012.12.001

**Published:** 2013-10

**Authors:** Andrew A. Crawford, Glyn Lewis, Sarah J. Lewis, Marcus R. Munafò

**Affiliations:** a School of Social and Community Medicine, University of Bristol, Oakfield House, Oakfield Grove, Bristol BS8 2BN, UK; b School of Experimental Psychology, University of Bristol, BS8 1TU, UK

**Keywords:** 5-HTTLPR, Antidepressant, SSRI, Meta-analysis, Discontinuation

## Abstract

There is evidence that 5-HTTLPR is associated with response following treatment from selective serotonin reuptake inhibitors (SSRIs). The short (S) allele has reduced serotonin transporter expression, compared to the long (L) allele, and has been reported to be associated with poorer response in Europeans, with the effect in other populations unclear. However the published literature is inconsistent. A systematic review and meta-analysis was performed to investigate the effect of 5-HTTLPR on discontinuation from antidepressant treatment. Data were obtained from 17 studies including 4309 participants. The principal outcome measure was the allelic odds ratio (*OR*) for the 5-HTTLPR S allele and discontinuation status. A random effects meta-analysis provided no evidence that the S allele was associated with increased odds of discontinuation from SSRIs in Europeans (*OR* 1.09, 95% CI 0.83–1.42, *p*=0.53; 10 studies, *n*=2504) but in East Asians there was evidence of a reduced odds of discontinuation (*OR* 0.28, 95% CI 0.12–0.64, *p*=0.002; 2 studies, *n*=136). There was a suggestion of small study bias (*p*=0.05). This meta-analysis provides no evidence of an association between 5-HTTLPR and discontinuation from antidepressant treatment in Europeans. The low number of studies in East Asian samples using SSRIs reduces confidence in our evidence that the S allele decreases the odds of discontinuation in this population. At present, there is no evidence of an association between 5-HTTLPR and discontinuation from SSRI treatment in a European population with further studies required to investigate its effects in different populations.

## 1 Introduction

Antidepressants are frequently prescribed in the treatment of depression. However, long-term treatment is required for antidepressants to successfully alleviate the symptoms of depression. Individuals who stop antidepressant treatment prematurely may not benefit from treatment and are at a higher risk of relapse (Montgomery et al., 1993; Donoghue et al., 1996). The most common reasons for early discontinuation from antidepressant treatment are adverse effects and lack of efficacy (Demyttenaere et al., 2001). It is not currently possible to accurately predict who will discontinue treatment. Variability in antidepressant response has been shown to be influenced by both genetic and environmental factors (Uher, 2008) creating the possibility of using genetic biomarkers capable of predicting discontinuation from treatment.

Selective serotonin reuptake inhibitors (SSRIs) are the first choice antidepressant due to their superior adverse effect profile. SSRIs increase the levels of serotonin in the synaptic cleft by binding to the serotonin reuptake transporter (5-HTT), preventing the reuptake and subsequent storage or degradation of serotonin. This leads to the accumulation of serotonin in the synaptic cleft, which in turn may cause adaptive changes in both serotonergic and noradrenergic neurotransmission and downstream neural adaptation, helping to alleviate the symptoms of depression (Hashimoto, 2009; Vidal et al., 2009). SSRI-induced side effects may occur when levels of synaptic serotonin increase to an intolerably high concentration resulting in over-stimulation of serotonin receptors in the brain and periphery (Ferguson, 2001).

The serotonin transporter gene-linked polymorphic region (5-HTTLPR) modulates transcriptional activity of 5-HTT. 5-HTTLPR is a 44 base pair insertion–deletion polymorphism which can exist as a long (L) variant of a 16 repeat sequence or a short (S) variant of 14 repeats. The L allele is associated with higher levels of transcription *in vitro* compared to the S allele (Lesch et al., 1996). Therefore, the same dose of SSRI may inhibit a higher proportion of 5-HTT in individuals carrying the S allele, causing a rapid accumulation of synaptic serotonin and increasing the risk of adverse effects, potentially leading to discontinuation. As studies have associated 5-HTTLPR with mood disorder (Bellivier et al., 1998; Hauser et al., 2003; Joiner et al., 2003) and unipolar depression (Clarke et al., 2010) it is important to distinguish between genuine pharmacogenetic effects as opposed to effects which simply reflect genotype acting as a marker for disease severity.

The association between 5-HTTLPR and antidepressant treatment has been subject to numerous studies with the majority investigating the outcome of response. In general, data on the number of discontinuations is collected but rarely published with regards to 5-HTTLPR. Murphy et al. (2004) found that discontinuation rates due to adverse effects were lower in patients of European ancestry receiving paroxetine who were L/L homozygotes. Several studies have reported that patients with an S allele more frequently experience adverse effects during treatment with SSRIs than L allele carriers (Perlis et al., 2003; Maron et al., 2009; Kato and Serretti, 2010). The largest study to date, using the STAR⁎D cohort, reported that a lesser burden of adverse effects from citalopram treatment was associated with the L allele (Hu et al., 2007). However, the authors reported no evidence of an association between 5-HTTLPR and intolerance (discontinuation with high adverse effect score) to citalopram. The second largest study to date, using the Genome Based Therapeutic Drugs for Depression (GENDEP) cohort, found no evidence of an association between 5-HTTLPR genotype and adverse effects, self-reported adherence or discontinuation with escitalopram or nortriptyline (Huezo-Diaz et al., 2009). Other studies have also failed to find evidence of an association between 5-HTTLPR variants and adverse reactions induced by various SSRIs including fluvoxamine (Takahashi et al., 2002; Kato et al., 2006), paroxetine (Kato et al., 2005; Tanaka et al., 2008) and sertraline (Ng et al., 2006) or have even reported the SS genotype to be associated with lower rates of agitation compared to those with SL/LL genotype (Kronenberg et al., 2007).

These contradictory findings have possibly occurred because studies *in vivo* have not consistently reported the L allele to be associated with an increase in transporter binding sites (Murthy et al., 2010). Other polymorphisms have also been reported to influence gene expression, in particular a single nucleotide polymorphism within the L allele (rs25531). This L_G_ allele may be associated with reduced transporter expression, in a similar manner to the S allele (Hu et al., 2006). Additionally, the role of ancestry may be important. There is a much higher frequency of the S allele in East Asian (79%) than in European (42%) populations (Kunugi et al., 1997). The difference in allele frequency has the potential to introduce confounding by population structure, as well as reducing the power in studies where the allele frequency is lower. In addition differences in linkage disequilibrium patterns between populations may be important if the SNP which is being studied is a proxy for the one which is influencing outcome.

There have been several meta-analyses attempting to clarify the role of 5-HTTLPR in response to antidepressant treatment. The most recent meta-analysis, which included 33 studies (5479 subjects), concluded that in Europeans 5-HTTLPR may be a predictor of antidepressant response and remission, while in East Asians it does not appear to play a major role (Porcelli et al., 2012). An earlier meta-analysis which included 28 studies (5408 subjects) concluded that the 5-HTTLPR bi-allelic short/long polymorphism by itself does not seem to predict antidepressant response to a clinically useful degree (Taylor et al., 2010). These conflicting findings may be due to the inclusion of different studies as well as stratifying by different factors. A meta-analysis of 9 studies with 2642 participants found that the L allele was associated with a reduced risk of experiencing side effects (Kato and Serretti, 2010).

To build on the work of previous meta-analyses we decided to investigate the association between 5-HTTLPR and the number of individuals who discontinue antidepressant treatment. Our outcome of discontinuation includes individuals who discontinued antidepressant treatment for any reason. We chose our outcome of discontinuation as it does not require an individual to make a potentially complex psychosocial judgement on the reason of discontinuation. Additionally, examining discontinuation is often used to study comparative acceptability of medication (Cipriani et al., 2009). Ideally our hypothesis would be tested by studying rates of adverse effects but unfortunately not all studies collect this data and exclusion of these studies could introduce bias. Our choice of outcome is clinically important as individuals who discontinue prematurely from antidepressant treatment are unable to benefit from the treatment and are at greater risk of relapse (Montgomery et al., 1993; Donoghue et al., 1996). To our knowledge this is the first meta-analysis to use an outcome of discontinuation from antidepressant treatment.

## 2 Experimental Procedures

### 2.1 Selection of studies for inclusion

Studies in which depressed individuals received antidepressant medication and data on discontinuations were reported by 5-HTTLPR polymorphism status were included. The principal outcome measure was the allelic odds ratio (*OR*) for the 5-HTTLPR S allele and discontinuation status. Ancestry was coded as European, East Asian, or other. If the study reported results by ancestry or was a randomised controlled trial (RCT), each population or randomised group was treated as a separate sample.

### 2.2 Search strategy

Electronic databases were searched (Embase, Web of Science and Medline) from the first date available in each database up to 18 September 2012 using the search terms “5-HT”, “5-HTT”, “SERT”, “serotonin”, “transporter”, “SLC6A4”, “5-HTTLPR”, “antidepressant”, “SSRI”, “SRI” or “reuptake inhibitor”. The full search strategy is available in Supplementary Figure 1. Studies were also identified from earlier reviews. Bibliographies of the collected articles were then hand-searched for additional references. The abstracts of the identified studies were then examined with reference to the inclusion and exclusion criteria by two researchers independently. Studies not written in English were translated by a native speaker. Data extraction was also performed by two individuals independently. In cases of disagreement over study eligibility or extraction of data, issues were discussed and a consensus reached. Studies with overlapping patient samples were identified and only the publication with the larger number of patients was included. Authors of published studies were contacted when the necessary data for this meta-analysis were not reported, and included if they provided the data.

### 2.3 Data analysis

Data were analysed within a random effects (DerSimonian-Laird) model and the individual and pooled *ORs* and associated 95% confidence intervals (*CI*) were calculated. Random-effects models are more conservative than fixed-effects models and assume that the true effect size could vary from study to study. The significance of the pooled *OR* was determined using a *Z*-test, and heterogeneity between studies was assessed with chi-squared goodness of fit and the *I*^*2*^ statistic. The *I*^*2*^ statistic quantifies the percentage of total variation across studies due to heterogeneity rather than chance variation. The higher the *I*^*2*^ statistic, the more between-study heterogeneity, with the values of 25%, 50%, and 75% considered as low, moderate, and high heterogeneity, respectively (Higgins et al., 2003). High between-study heterogeneity indicates potential methodological or sample differences. Our primary analysis compared the effect of the S allele with the L allele on discontinuation, but given the lack of unequivocal data for the 5-HTTLPR genetic model, all models were tested: S allele vs. L allele, S carriers vs. LL, L carriers vs. SS, and SS vs. LL. If data were available, a secondary analysis was performed with an outcome of discontinuation due to adverse effects. We conducted a series of stratified analyses and meta-regression analyses to assess the impact of various study characteristics, including class of antidepressant (SSRI vs. non-SSRI), ancestry (European vs. East Asian vs. Other), year of publication, mean sample age, percentage male, study size and study duration. The genotype distributions for each study were used to calculate deviation from Hardy–Weinberg equilibrium (HWE) using a *χ*^2^ test, and those studies which did show evidence of deviation were excluded in a sensitivity analysis. An influence analysis was also performed to investigate the effects of excluding single studies. To assess potential small study bias, as might be caused by publication bias, a funnel plot was prepared of ln *OR* against the *SE* ln *OR* (Sterne and Egger, 2001) and analysed using Egger's test (Egger et al., 1997). An asymmetric funnel plot indicates a relationship between treatment effect and study size. Data were analysed using Stata version 12.1 (StataCorp, 2011). We report exact *p*-values throughout.

## 3 Results

### 3.1 Description of studies

A total of 17 studies (longitudinal studies (*n*=11) and RCT's (*n*=6)) (Joyce et al., 2003; Durham et al., 2004; Murphy et al., 2004; Kato et al., 2005; Kato et al., 2006; Hu et al., 2007; Kang et al., 2007a; Kang et al., 2007b; Serretti et al., 2007; Dogan et al., 2008; Gressier et al., 2009; Higuchi et al., 2009; Huezo-Diaz et al., 2009; Ruhe et al., 2009; Saeki et al., 2009; Yoshimura et al., 2009; Gudayol-Ferre et al., 2010; Illi et al., 2010; Lanctot et al., 2010; Safarinejad, 2010; Umene-Nakano et al., 2010; Yuksel et al., 2010; Illi et al., 2011; Lewis et al., 2011; StataCorp, 2011), comprising *k*=22 independent samples (each arm of the RCT was treated as a separate sample if the interventions were pharmacologically different and not placebo. Different ancestries were also treated as separate samples) published between 2003 and 2011, were identified by the search strategy and included in the meta-analysis (Supplementary Figure 2). A total of 4309 individuals (848 discontinuations) were included and samples varied in size from 30 to 980 individuals. The characteristics of these samples are presented in Table 1. Of these, *k*=15 samples were derived from populations of predominantly European ancestry, *k*=4 from populations of East Asian ancestry, and *k*=3 from other populations. The study by Kato et al. (2006) only provided genetic data comparing SS homozygotes with L allele carriers and so had to be excluded from other analyses. All studies included individuals with a diagnosis of depression, however one study looked at depressed patients after brain trauma (Lanctot et al., 2010), and one study also included individuals with bipolar disorder (Serretti et al., 2007). The study by Serretti et al. (2007) and a sample from the study by Hu et al. (2007) deviated from HWE (*p*=0.03 and *p*<0.001, respectively).

### 3.2 Meta-analysis

#### 3.2.1 Exploratory analysis

A total of 22 independent samples (4309 individuals in total: The “SSRI” antidepressant analysis included 16 samples and 3311 individuals; the “other” antidepressants analysis included 6 samples and 998 individuals) were included. We found no evidence that the S allele was associated with discontinuation from antidepressant treatment overall (*OR* 0.95, 95% CI 0.79–1.14, *p*=0.55) or in the sub-group analyses in the SSRI group (*OR* 0.98, 95% CI 0.77–1.24, *p*=0.83) or other antidepressant group (*OR* 0.88, 95% CI 0.72–1.09, *p*=0.24). There was evidence of moderate heterogeneity (*χ*^*2*^_[20]_=41.67, *p*=0.003, *I*^*2*^=52%) which remained when the SSRI group was considered separately (*χ*^*2*^_[14]_=34.65, *p=*0.002, *I*^*2*^=60%). Analysis by different genotype models did not substantially alter the results (results available on request). Excluding any individual study, any study not in HWE (Hu et al., 2007; Serretti et al., 2007), or any study including depressed individuals with another diagnosed disorder (Serretti et al., 2007; Lanctot et al., 2010), did not alter our results substantially.

#### 3.2.2 Analysis in European individuals

**Figure 1:**
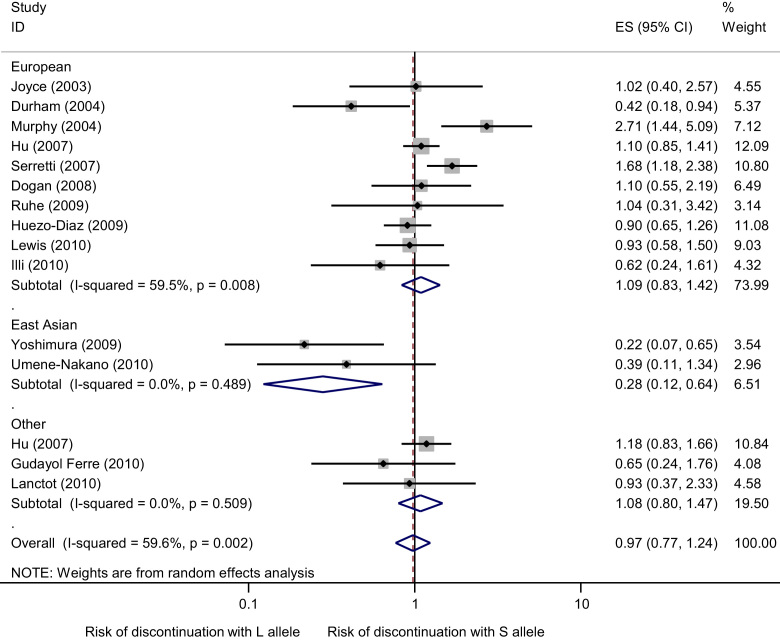
Meta-analysis of association studies of 5-HTTLPR genotype and SSRI discontinuation stratified by ancestry (European vs. East Asian vs. Other). Meta-analysis provides no evidence of an association between 5-HTTLPR genotype and SSRI discontinuation for European studies (*z*=0.63, *p*=0.53) for other studies (*z*=0.51, *p*=0.61), or overall (*z*=0.21, *p*=0.8). There is evidence of an association between 5-HTTLPR genotype and SSRI discontinuation for East Asian studies (*z*=3.03, *p*=0.002). Bars represent individual study 95% confidence intervals, with a central block proportional to study size. The summary diamond bars represent the pooled effect size estimate and 95% confidence interval (CI) for the European studies, East Asian studies, other studies and all studies, as you look from top to bottom.

A total of 15 independent samples (3367 individuals in total: 10 samples and 2504 individuals included in the “SSRI” antidepressant analysis; 5 samples and 863 individuals included in the “other” antidepressants analysis) were included. We found no evidence that the S allele was associated with increased odds of discontinuation from antidepressant treatment overall (*OR* 1.00, 95% CI 0.81–1.22, *p*=0.96) or in the SSRI group (*OR* 1.09, 95% CI 0.83–1.42, *p*=0.53) or other antidepressant group (*OR* 0.86, 95% CI 0.68–1.09, *p*=0.22). There was evidence of moderate heterogeneity (*χ*^*2*^_[14]_=30.14, *p*=0.007, *I*^*2*^=54%) which remained when the SSRI group was considered separately (*χ*^*2*^_[9]_=22.22, *p*=0.008, *I*^*2*^=60%). These results are displayed graphically in Figure 1. Analysis by different genotype models did not substantially alter the results (Supplementary Figures 3–5). Excluding any individual study, any study not in HWE or any study not exclusively of depressed individuals did not alter our results substantially.

#### 3.2.3 Analysis in East Asian individuals

A total of 4 independent samples (371 individuals in total: 3 samples and 236 individuals included in the “SSRI” antidepressant analysis; 1 sample and 135 individuals included in the “other” antidepressants analysis) were included. The study by Kato et al. (2006) only provided genetic data in a format that allowed inclusion in the analysis comparing SS homozygotes with L allele carriers (SS vs. SL/LL). We found evidence that the S allele (S vs. L) was associated with reduced odds of discontinuation from SSRI treatment (*OR* 0.28, 95% CI 0.12–0.64, *p*=0.002; Figure 1). Comparing homozygote individuals (SS vs. LL) did not alter results substantially. There was weak statistical evidence of an association when comparing SS homozygotes with L allele carriers (*OR* 0.34, *p*=0.08), and when comparing LL homozygotes with S allele carriers (LL vs. SL/SS) (*OR* 0.24, *p*=0.14).

#### 3.2.4 Discontinuation due to adverse effects

We investigated an outcome of discontinuations specifically due to adverse effects in Europeans receiving SSRI treatment (5 independent samples (Murphy et al., 2004; Hu et al., 2007; Dogan et al., 2008; Ruhe et al., 2009; Illi et al., 2010), 1307 individuals were included). A per allele analysis found no evidence of an association (OR 1.43, 95% CI 0.74–2.76, *p=*0.3; data available by request).

#### 3.2.5 Small study bias

Visual inspection of a funnel plot of all studies indicated asymmetry, suggesting that some small studies reporting an association between the S allele and increased risk of discontinuation may be missing (Supplementary Figure 6). Egger's test provided some evidence of small study bias (*p*=0.05) when all studies were included, although this test has low statistical power given the small number of studies. Evidence of small study bias no longer remained when only European studies were included (*p*=0.34). There was no evidence between individual study effect size estimate and year of publication (*p*=0.55), mean sample age (*p*=0.91), study size (*p*=0.32), percentage male (*p*=0.55) or study duration (*p*=0.30) when all studies were included, or when restricted to European only studies (results available on request).

## 4 Discussion

To our knowledge, this is the first meta-analysis to assess the association of 5-HTTLPR genotype with discontinuation following SSRI treatment. Our results find no evidence of an association between 5-HTTLPR and discontinuation from SSRI treatment in a European population. This supports the findings from the three largest studies on a predominantly European population (STAR*D (Hu et al., 2007), GENDEP (Huezo-Diaz et al., 2009) and Genetic and clinical Predictors Of treatment response in Depression (GENPOD) (Lewis et al., 2011)), suggesting that there is no evidence of an association between the bi-allelic 5-HTTLPR polymorphism with SSRI discontinuation in Europeans. However, due to the low number of discontinuation events we are unable to rule out the possibility of a clinically important effect with SSRI treatment as the S allele may be associated with up to a 42% increase in odds of discontinuation or a 17% reduced odds (*OR* 1.09, 95% CI 0.83–1.42).

We found some evidence that the S allele decreases the odds of discontinuation from SSRI treatment in East Asians. The low number and small size of East Asian studies (only 17 dropouts in the SSRI analysis) included in this meta-analysis mean that further studies are required to investigate its effects before we can draw any firm conclusions. There was evidence of considerable heterogeneity even within Europeans treated with an SSRI. The cause of this heterogeneity is unknown as there was substantial consistency in the diagnostic criteria as well as antidepressant agents used.

This meta-analysis investigated the 5-HTTLPR as a bi-allelic polymorphism only. Ideally polymorphisms such as rs25531 and VNTR STin2 should be included as these have also been reported to affect gene expression (Hranilovic et al., 2004), although the functionality of some of these variants remains uncertain (Parsey et al., 2006). Other polymorphisms were not included in this meta-analysis as not all studies have the necessary genetic data. From our 17 studies only 6 had the necessary genetic data for rs25531, and exclusion of the other studies would result in loss of power and could introduce bias. The largest study to date reported an analysis in which individuals were differentiated according to the presence of the L_A_ allele, this stratification did not substantially alter results when analysing the whole STAR*D sample. However, it did alter the results of a sub-group analysis of white, non-Hispanic individuals with adverse effect burden as the outcome (Hu et al., 2007).

Commonly cited reasons for discontinuing antidepressant treatment are due to adverse effects or lack of efficacy. The evidence from the two most recent meta-analyses investigating these outcomes (adverse effects (Kato and Serretti, 2010), response (Porcelli et al., 2012)) suggests that the L allele is associated with a reduced risk of experiencing adverse effects, and with increased odds of responding to SSRI treatment. However, we found no evidence for an association between the L allele and reduced odds of discontinuation. There are multiple reasons why an individual may choose to stop antidepressant treatment, not associated with antidepressant response or adverse effects, such as fear of drug dependency. Therefore, our outcome of all discontinuations may have been too broad to find evidence of an association with 5-HTTLPR. However, our analysis of discontinuation due to adverse effects also found no evidence of an association with 5-HTTLPR. The low number of independent samples with data on reason of discontinuation mean this analysis lacked the power to detect a potentially important effect and further work could focus on discontinuation specifically due to adverse effects or lack of efficacy.

As mentioned previously the low number of Asian studies included in this meta-analysis mean we are currently unable to determine the effect of this polymorphism in an East Asian population. However, the results of this meta-analysis summarise the available data on SSRI-discontinuation and 5-HTTLPR in a European population. At present, there is no evidence of an association between 5-HTTLPR and discontinuation from SSRI treatment in a European population. The lack of precision in our effect estimate (indicated by our wide 95% CI) highlights the need for greater research in this area before we can make any definite conclusions on its potential clinical importance. A meta-analysis of approximately 5400 European individuals would be required to determine whether 5-HTTLPR has at least a 15% change in risk of discontinuation from SSRI treatment. Future studies should report adverse effects and discontinuation alongside the more customary efficacy measurements in order to improve our knowledge of the 5-HTTLPR and its effect on outcomes following antidepressant treatment.

## Role of funding source

AC is funded by the Wellcome Trust. The Wellcome Trust had no further role in the study design; in the collection, analysis and interpretation of data; in the writing of the report; and in the decision to submit the paper for publication.

## Contributors

AC performed the electronic search which was checked in duplicate by GL, SL or MM. All authors contributed to the drafting of the review.

## Conflict of Interest

There are no relevant conflicts of interest to declare.

## Figures and Tables

**Table 1:** Characteristics and summary of results of included samples.

**Sample**	**Year**	**Sample size**	**No. of dropouts**	**SS (%)**	**Antidepressant**	**Ancestry**	**Mean Age**	**Male (%)**	**HWE**
Joyce (38)	2003	86	11	34	Fluoxetine	European	32	44	Yes
Joyce (38)	2003	82	25	27	Nortriptyline	European	32	44	Yes
Murphy (13)	2004	121	30	20	Paroxetine	European	72	47	Yes
Murphy (13)	2004	123	18	25	Mirtazapine	European	72	50	Yes
Durham (36)	2004	101	20	16	Sertraline	European	70	47	Yes
Kato (20)	2006	100	20	60	Paroxetine, Fluvoxamine	East Asian	44	56	Yes
Hu (17)	2007	420	64	21	Citalopram	Other	42	39	No
Hu (17)	2007	980	137	18	Citalopram	European	42	39	Yes
Kang (48)	2007	135	34	76	Mirtazapine	East Asian	50	29	Yes
Serretti (44)	2007	281	86	24	Fluvoxamine, Paroxetine, Sertraline	European	49	32	No
Dogan (39)	2008	64	20	33	Sertraline	European	37	22	Yes
Yoshimura (42)	2009	71	11	35	Paroxetine	East Asian	44	44	Yes
Huezo-Diaz (18)	2009	450	92	17	Escitalopram	European	43	38	Yes
Huezo-Diaz (18)	2009	345	94	15	Nortriptyline	European	42	35	Yes
Ruhe (43)	2009	51	7	16	Paroxetine	European	42	36	Yes
Lewis (35)	2010	273	41	19	Citalopram	European	39	36	Yes
Lewis (35)	2010	283	93	17	Reboxetine	European	39	34	Yes
Illi (37)	2010	97	12	13	Citalopram, Fluoexetine, Paroxetine	European	42	42	Yes
Yuksel (41)	2010	30	10	27	Venlafaxine	European	37	43	Yes
Umene-Nakano (45)	2010	65	6	60	Sertraline	East Asian	55	37	Yes
Lanctot (46)	2010	79	9	30	Citalopram	Other	40	56	Yes
Gudayol Ferre (47)	2010	72	8	39	Fluoxetine	Other	31	8	Yes

Characteristics of included samples is presented, with year, number of subjects, number of discontinuations, percentage (%) of individuals homozygous with the short (S) 5-HTTLPR allele, type of antidepressant, sample ancestry, mean age, percentage of males in the sample, and whether the genotype frequencies reported were in approximate Hardy–Weinberg Equilibrium (HWE).

## Footnotes

Appendix A Supplementary data associated with this article can be found in the online version at doi:10.1016/j.euroneuro.2012.12.001.
