# Supplementary material for: Systematic review and meta-analysis of serotonin transporter genotype and discontinuation from antidepressant treatment
Source: Eur Neuropsychopharmacol. 2013 Oct;23(10):1143–50. doi: 10.1016/j.euroneuro.2012.12.001 (PMC3791403; doi:10.1016/j.euroneuro.2012.12.001)
Supplement: Supplementary file 1 — Supplementary Material [file mmc1.docx]

Supplementary Figure 1. Embase search strategy

1. exp serotonin transporter/

2. (Serotonin adj3 transporter).tw.

3. sert.tw.

4. 5htt.tw.

5. 5-htt.tw.

6. serotonin transporter.tw.

7. 5ht transporter.tw.

8. 5-ht transporter.tw.

9. gene*.tw.

10. polymorphi*.tw.

11. or/1-8

12. or/9-10

13. 11 and 12

14. 5httlpr.tw.

15. 5-HTTLPR.tw.

16. Slc6a4.tw.

17. or/13-16

18. exp Antidepressive Agents/

19. exp Neurotransmitter Uptake Inhibitors/

20. exp Monoamine Oxidase Inhibitors/

21. (antidepress* or anti-depress* or pharmacotherap* or psychopharma* or psycho-pharma* or MAOI or monoamine oxidase inhibit* or ((serotonin or norepinephrine or noradrenaline or nor-epinephrine or nor-adrenaline or neurotransmitter* or dopamine*) and (uptake or reuptake or re-uptake)) or SSRI* or SNRI* or TCA* or tricyclic* or tetracyclic*).mp.

22. (citalopram or Escitalopram or fluoxetine or Fluvoxamine or Paroxetine or sertraline).mp. [mp=title, abstract, subject headings, heading word, drug trade name, original title, device manufacturer, drug manufacturer, device trade name, keyword]

23. or/19-23

24. 18 and 24

Supplementary Figure 2. Study flow


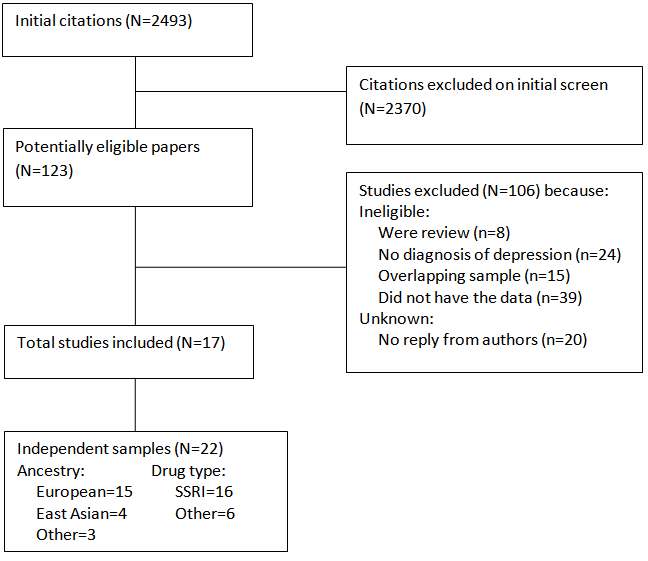


Figure showing the initial number of citations through to the number of studies and samples included in the meta-analysis.

Supplementary Figure 3. Outcome data for S carrier versus LL genotype, stratified by ancestry (European v. East Asian v. Other)

Meta-analysis provides no evidence of an association between 5-HTTLPR genotype and SSRI discontinuation for European studies (z = 0.07, *p* = 0.94), East Asian studies (z = 1.49, *p* = 0.14), other studies (z = 0.40, *p* = 0.69) or overall (z = 0.55, *p* = 0.69).

Supplementary Figure 4. Outcome data for L carrier versus SS genotype, stratified by ancestry (European v. East Asian v. Other)

Meta-analysis provides no evidence of an association between 5-HTTLPR genotype and SSRI discontinuation for European studies (z = 1.65, *p* = 0.10), East Asian studies (z = 1.71, *p* = 0.09) other studies (z = 0.46, *p* = 0.64) or overall (z = 0.69, *p* = 0.49).

Supplementary Figure 5. Outcome data for LL genotype versus SS genotype (excluding SL heterozygotes), stratified by ancestry (European v. East Asian v. Other)

Meta-analysis provides no evidence of an association between 5-HTTLPR genotype and SSRI discontinuation for European studies (z = 1.11, *p* = 0.27) other studies (z = 0.50, *p* = 0.62) or overall (z = 0.48, *p* = 0.63) but there is evidence for East Asian studies (z = 2.42, *p* = 0.015).

Supplementary Figure 6. Funnel plot of log OR against standard error log OR (SSRI studies)

Funnel plot of SSRI studies suggests asymmetry which could be corrected by the addition of small studies reporting an association of S allele with SSRI discontinuation.
